# Prevalence and clinical correlates of self-harm and suicidality during admission of children in a mental health inpatient unit

**DOI:** 10.1192/j.eurpsy.2020.108

**Published:** 2020-12-16

**Authors:** Eleftherios Kipoulas, Azi Berzengi, Marinos Kyriakopoulos

**Affiliations:** 1 National and Specialist Acorn Lodge Inpatient Children’s Unit, Child and Adolescent Mental Health Services, South London and the Maudsley NHS Foundation Trust, London, United Kingdom; 2 Cambridgeshire and Peterborough NHS Foundation Trust, Cambridge, United Kingdom; 3 Department of Child and Adolescent Psychiatry, Institute of Psychiatry, Psychology and Neuroscience, King’s College London, London, United Kingdom

**Keywords:** Children, inpatient admission, mental health, self-harm, suicidality

## Abstract

**Background:**

Self-harm and suicidality are common presentations in children and adolescents requiring a mental health inpatient admission. Although there are several studies on adolescents, there is relatively limited research into childhood self-harm and suicidality during such admissions.

**Methods:**

A retrospective electronic file review was conducted on all children discharged from a national mental health inpatient children’s unit over a 6-year period. Several independent variables were compared between self-harm/suicidal and non-self-harm/non-suicidal children. Separate analyses investigated changes in self-harm/suicidality, functional outcomes, and risk assessment ratings between admission and discharge.

**Results:**

A total of 105 children were included in this study. During admission, 65.7% of them reported self-harm thoughts, 61% engaged in self-harm, 50.5% expressed suicidal thoughts, and 14.3% engaged in suicidal behavior. Thoughts and acts of self-harm were associated with previous self-harm, longer admissions, and Attention Deficit Hyperactivity Disorder. Suicidality overlapped with self-harm and was strongly predicted by previous self-harm. The prevalence of self-harm and suicidal thoughts and acts significantly decreased during admission. Children in the non-self-harm group had marginally better functional outcomes upon discharge compared to those in the self-harm group. Children and parents/caregivers were similarly satisfied with treatment, irrespective of children’s self-harm/suicidality.

**Conclusions:**

Self-harm and suicidality were widespread among children admitted to hospital. Better understanding of the mechanisms and factors related to self-harm and suicidality in this age group could help mitigate associated risks and improve existing safety strategies.

## Introduction

Self-harm and suicidality among children and young people are increasingly recognized as a major public health concern. Although completed suicide is relatively rare in this age group, it is still the second, after accidents, most common cause of death in adolescence in most developed countries [1], with a worldwide pooled mortality rate of 3.77/100,000 among youths [2]. National reports from the Youth Risk Behavior Surveillance System in the USA showed that 17.2% of the high school students reported suicidal ideation, 13.6% had a plan to die, and 7.4% attempted suicide in the past 12 months [3]. In addition, the 12-month prevalence of self-harm without intent to die in high school students was found to range from 6.4 to 14.8% for boys and 17.7 to 30.8% for girls across 11 different U.S. states [4]. Similar findings on self-harm were reported in Europe, with a reported lifetime prevalence of 28% in school pupils in 11 European countries [5], and an estimated lifetime prevalence of 16% in adolescents in England [6].

Although accurate prevalence rates of self-harm and suicidality in childhood are more difficult to estimate, such presentations are thought to be relatively rare before the age of 12 in community samples [7–13]. However, even in this age range, their prevalence is not insignificant; a recent study evaluating a large representative community cohort of 7,994 children with mean age of 9.9 years, identified 8.4% of children reporting past or current suicidal ideation, 0.9% past or current suicidal plans, and 1.3% past or current suicidal attempts [14]. In clinical samples, these figures are much higher [15–23]. Self-harm and suicidality frequently co-occur with a range of mental and social problems [14,24,25] and have been found to be associated with adverse family relationships and educational factors [14,24,26–31]. Nevertheless, many gaps remain in the current knowledge on the prevalence and clinical correlates of self-harm and suicidality in youth clinical populations [2,24,28,31–35].

A large proportion of children and young people requiring inpatient mental health treatment present with self-harm and suicidality. DiClemente and colleagues [36] found that 61% of admitted adolescents engaged in scratching or cutting behavior. In another similar sample, 25% harmed themselves, 13.4% attempted suicide, and 30.9% engaged in both self-harm and suicide attempts [16]. In studies with mixed samples of children and adolescents, self-harm thoughts appeared to be more frequent before the age of 12, but adolescents were more likely to engage in self-injurious behavior [18,21]. However, suicidality appears to be very common even among children younger than 13 years who require admission, with an estimated prevalence of reported suicidal ideation as high as 42.9%, and a reported suicide attempt rate of 4.2% in a Finish national sample [37].

Several studies highlight the importance of specifically investigating childhood self-harm and suicidality. Childhood self-harming behavior or suicidal ideation reliably predict self-harm at the age of 15 [30] and later suicide [38–40]. Furthermore, Zanarini and colleagues [41], in their investigation of inpatient adults with Borderline Personality Disorder, found that those who started self-harming in childhood had been engaging in such behaviors for longer. Childhood-onset self-harm is also associated with a wider range of self-injurious means compared to self-harm starting in adolescence or adulthood [41]. Finally, children who were admitted as medical emergencies for self-harm or suicidal behavior had more complex mental health needs than those referred for psychiatric help for other reasons [27,42–46].

Research focusing on self-harm and suicidality among hospitalized children is limited. Some studies have examined mixed samples of child and adolescent inpatients [18,47], or not included comparisons with children without these complaints [44]. In addition, suicidal risk seems to have incorporated self-harm thoughts and behaviors [48–50], or suicidality seems to have been investigated at the exclusion of self-harm [37,51]. To the best of our knowledge, there have not been any studies that explore separately both self-harm and suicidality including comparison groups in this group of children. In our study, we aimed to investigate the prevalence and clinical correlates of self-harm and suicidality in children aged 6–12 years requiring inpatient mental health treatment, also generating hypotheses regarding potential predictors of self-harm and suicidality during such admissions.

## Methods

### Case definition of self-harm and suicidality

We used the term “self-harm” to describe thoughts and acts that were not associated with wishing or intending to end one’s life [52]. By contrast, we defined suicidal thoughts and acts by the presence of such a wish or intent [53,54].

Children who expressed self-harm thoughts or engaged in at least one self-injurious act during their admission were allocated to the “self-harm group,” while those who did not were allocated to the “non-self-harm group.” Similarly, children who presented with suicidal thoughts or acts were allocated to the “suicidal group,” while those who did not were allocated to the “non-suicidal group.” The “self-harm” and “suicidal” groups were not mutually exclusive; children could potentially be allocated to both groups if they presented with both self-harm and suicidal thoughts and acts.

### Measures and procedure

All children discharged between January 2013 and April 2019 from our inpatient mental health unit whose inpatient admission was longer than 2 weeks were included. We employed a retrospective, descriptive cohort and case-control investigation of the extent of self-harm/suicidal thoughts and acts, which were our outcome variables. Sociodemographic and clinical information were extracted from electronic records. Psychiatric diagnoses were determined according to the Multiaxial ICD-10 classification system of psychiatric disorders in childhood and adolescence [55] and the ICD-10 diagnostic criteria for research [56].

Self-harm/suicidal thoughts and acts and predictor variables, including history of self-harm, family history of mental illness, and anomalous parenting situation (ICD-10), were coded on a dichotomous scale (Present/Absent). Length of admission for each child was divided into two equal parts to explore changes of self-harm/suicidality prevalence over time. Finally, self-harm and suicide risk assessment ratings on admission and at discharge were analyzed. These consisted of five levels of risk corresponding to ratings in each child’s structured risk assessment as low (1), low-moderate (2), moderate (3), moderate-high (4), and high (5). These were based on the clinicians’ judgment at the time of the assessment who were blinded to the purpose of this study.

Functional outcomes were assessed using the Children’s Global Assessment Scale (CGAS) [57] and the Health of the Nation Outcome Scales for Children and Adolescents (HoNOSCA) [58], administered on admission and at discharge. The CGAS measures global functioning and has values from 1, representing the lowest level of functioning, to 100, representing the highest. The HoNOSCA is a 15-item questionnaire with each item indicating the severity of a problem on a scale of 0–4 focusing on general health and social functioning [59].

Children and parent/carer satisfaction were measured using the Acorn Satisfaction Questionnaire (ASQ), a nine-item self-report measure (seven items for parents/carers and two items for children) which has been previously used with this population. Each item is rated on a Likert scale (1–5), with higher scores indicating higher satisfaction [60].

This study was part of a service evaluation project which was approved by the South London and Maudsley NHS Foundation Trust Child and Adolescent Mental Health Services Clinical Academic Group Clinical Governance/Audit Committee.

### Statistical analysis

Chi-square and independent groups *t*-test were conducted for categorical and continuous variables, respectively, using SPSS Statistics Version 24.0. Separate analyses were conducted between the self-harm and non-self-harm groups, and between the suicidal and non-suicidal groups. The prevalence of self-harm/suicidal thoughts and acts was compared between the first and second half of admission. Multiple binary logistic regression analyses were conducted to determine predictor variables of self-harm and suicidality based on the variables that were found to be significantly associated with these. Odds ratios (OR) are reported in statistically significant associations (*p* < 0.05).

### Missing data

The response rate for the ASQ children items was 76.2% and for the parent/carer items was 81.9%. CGAS scores were missing for one child (data available for 99%) and HoNOSCA scores for 10 children (data available for 90.5%).

## Results

### Clinical profile of participants

A total of 107 children were discharged from our unit between 2013 and 2019. Two admissions were excluded because they were less than 2 weeks long. Just under half of the sample (*n* = 50, 48%) were female and 72.4% (*n* = 76) were White. The mean age on admission was 10.5 years (SD = 1.5). The mean length of admission was 175 days (SD = 119, range: 25–704).

In 74.3% of the cases, children satisfied diagnostic criteria for more than one disorder. The most common diagnoses at discharge were anxiety disorders (61.9%), autism spectrum disorders (ASD; 57.1%), attention deficit hyperactivity disorders (ADHD; 35.4%), psychotic disorders (14.3%), mood disorders (13.3%), intellectual disability (ID; 12.4%), and eating disorders (10.5%).

On admission, 33.3% of the children were classified as low risk for self-harm, 27.6% as low/moderate risk, 22.9% as moderate risk, 10.5% as moderate/high risk, and 5.7% as high risk. Similarly, on admission 43.8% of the sample presented with low suicide risk, 33.3% with low/moderate risk, 8.6% with moderate risk, 8.6% with moderate/high risk, and 5.7% with high risk.

Of the included 105 children, 67.6% (*n* = 71) had family history of mental illness, 63.8% (*n* = 67) had engaged in self-injurious behavior prior to their admission, and 60% (*n* = 63) were classified as experiencing an anomalous parenting situation (Multiaxial ICD-10), most commonly not living with two biological parents.

### Prevalence of self-harm and suicidal acts during admission

Sixty-nine children (65.7%) reported self-harm thoughts and 64 children (61%) injured themselves at least once during their admission. Over half of our sample (*n* = 53, 50.5%) reported suicidal thoughts and 15 children (14.3%) engaged in at least one suicidal act. The demographic and clinical characteristics of the sample are detailed in Tables 1 and 2. The types of self-harm and suicidal acts during admission are detailed in Table 3. There were five children without known self-harm or suicidal thoughts before admission who presented with self-harm/suicidality during admission: two of them (with diagnoses of ADHD and psychosis/ID) developed self-harm/suicidal thoughts, two (with diagnoses of ASD and psychosis/ID) self-harmed, and one with an eating disorder developed both self-harm and suicidal thoughts and acts. All children who engaged in a suicidal act were also found to have engaged in self-harm.

**Table 1.** Demographic and clinical characteristics of self-harm group (*N* = 69) vs. non-self-harm group (*N* = 36).

Variable	Self-harm group	Non-self-harm group	Significance (two-tailed)	OR (95% CI)
Demographics				
Female gender *n* (%)	31 (44.9)	19 (52.8)	0.445	NS
White *n* (%)	53 (76.8)	23 (63.9)	0.160	NS
Age on admission *M* (SD)	10.5 (1.4)	10.4 (1.8)	0.894	NS
Social worker involvement *n* (%)	49 (71)	21 (58.3)	0.191	NS
Looked after children *n* (%)	3 (4.3)	4 (11.1)	0.228	NS
Length of admission *in days M* (SD)	195.1 (131.9)	136.4 (75.5)	0.015*	1.01* (1.00, 1.01)
Diagnosis *n* (%)				
Anxiety	45 (65.2)	20 (55.6)	0.333	NS
ASD	44 (63.8)	16 (44.4)	**0.058** ^**§**^	**2.20** ^**§**^ **(0.97, 5.0)**
ADHD	29 (42)	8 (22.2)	0.044*	2.54* (1.01, 6.37)
Depression	11 (15.9)	3 (8.3)	0.276	NS
Psychosis	10 (14.5)	5 (13.9)	0.933	NS
Eating disorders	5 (7.2)	6 (16.7)	0.135	NS
Intellectual disability	9 (13)	4 (11.1)	0.775	NS
Risk factors *n* (%)				
Previous self-farm	55 (79.7)	12 (33.3)	<0.001**	7.86** (3.17, 19.48)
Family history of mental illness	51 (73.9)	20 (55.6)	**0.056** ^**§**^	**2.27** ^**§**^ **(0.97, 5.30)**
Anomalous parenting situation	41 (59.4)	22 (61.1)	0.867	NS
CGAS score *M* (SD)				
Admission	29.6 (11.3)	30.5 (11.2)	0.700	NS
Discharge	55 (12.1)	64.6 (13.8)	<0.001**	0.94** (0.90, 0.98)
Change	25.4 (16.1)	34.1 (16.8)	0.011*	0.97* (0.94, 1.0)
HoNOSCA score *M* (SD)				
Admission	22 (6.4)	19.2 (7.3)	0.049*	1.07* (1.00, 1.14)
Discharge	11.9 (6.2)	8.7 (5.7)	0.014*	1.10* (1.02, 1.19)
Change	10 (7.1)	10.5 (6.8)	0.751	NS
Self-harm risk rating *M* (SD)				
Admission	2.6 (1.2)	1.7 (0.9)	<0.001**	2.06** (1.33, 3.19)
Discharge	2 (1.1)	1.2 (0.4)	<0.001**	4.87** (2.09, 11.37)
Suicidal thoughts *n* (%)	50 (72.5)	3 (8.3)	<0.001**	28.95** (7.93, 105.64)
ASQ score *M* (SD)				
Parents/carers	30.6 (4.8)	31.9 (3.9)	0.220	NS
Children	7.4 (2.6)	8.2 (2)	0.140	NS
Total	36.5 (7.4)	37.8 (9.2)	0.475	NS
Total	69 (65.7%)	36 (34.3%)		

Abbreviations: ADHD, Attention Deficit Hyperactivity Disorder; ASD, Autism Spectrum Disorder; ASQ, Acorn Satisfaction Questionnaire; CGAS, Children’s Global Assessment Scale; CI, confidence interval; HoNOSCA, Health of the Nation Outcome Scales for Children and Adolescents; M, mean; *n*, number; NS, not significant; OR, odds ratio; SD, standard deviation.

Statistical significance: ^§^trend level, *p < 0.05, **p ≤ 0.001.

**Table 2.** Demographic and clinical characteristics of the suicidal group (*N* = 53) vs. the non-suicidal group (*N* = 52).

Variable	Suicidal group	Non-suicidal group	Significance (two-tailed)	OR (95% CI)
Demographics				
Female gender *n* (%)	23 (43.4)	27 (51.9)	0.382	NS
White *n* (%)	42 (79.2)	34 (65.4)	0.112	NS
Age on admission *M* (SD)	10.5 (1.3)	10.5 (1.7)	0.970	NS
Social worker involvement *n* (%)	37 (69.8)	33 (63.5)	0.490	NS
Looked after children *n* (%)	3 (5.7)	4 (7.7)	0.716	NS
Length of admission in days *M* (SD)	188.6 (123.4)	161.1 (113.1)	0.237	NS
Diagnosis *n* (%)				
Anxiety	34 (64.2)	31 (59.6)	0.632	NS
ASD	32 (60.4)	28 (53.8)	0.499	NS
ADHD	20 (37.7)	17 (32.7)	0.589	NS
Depression	10 (18.9)	4 (7.7)	0.092	NS
Psychosis	5 (9.4)	10 (19.2)	0.151	NS
Eating disorders	4 (7.5)	7 (13.5)	0.322	NS
Intellectual disability	2 (3.8)	11 (21.2)	0.007**	0.15** (0.03, 0.70)
Risk factors *n* (%)				
Previous self-farm	42 (79.2)	25 (48.1)	0.001***	4.12*** (1.75, 9.73)
Family history of mental illness	40 (75.5)	31 (59.6)	0.083	NS
Anomalous parenting situation	34 (64.2)	29 (55.8)	0.381	NS
CGAS score *M* (SD)				
Admission	30.2 (9.9)	29.7 (12.4)	0.821	NS
Discharge	57.3 (13.3)	59.4 (13.7)	0.425	NS
Change	27.1 (16.9)	29.7 (16.7)	0.429	NS
HoNOSCA score *M* (SD)				
Admission	21.4 (6.3)	20.7 (7.4)	0.627	NS
Discharge	11 (6)	10.6 (6.4)	0.696	NS
Change	10.2 (7.1)	10.2 (6.8)	0.987	NS
Suicide risk rating *M* (SD)				
Admission	2.3 (1.3)	1.7 (1)	0.010**	1.59** (1.10, 2.30)
Discharge	1.6 (0.8)	1.2 (0.4)	0.001***	3.32*** (1.58, 6.99)
Self-harm thoughts *n* (%)	50 (94.3)	19 (36.5)	<0.001***	28.95*** (7.93, 105.60)
Self-harm acts *n* (%)	47 (88.7)	18 (34.6)	<0.001***	16.13*** (5.77, 45.10)
ASQ score *M* (SD)				
Parents/carers	30.9 (4.4)	31.2 (4.6)	0.740	NS
Children	7.2 (2.5)	8.2 (2.2)	0.072	NS
Total	36.4 (7.4)	37.5 (8.6)	0.527	NS
Total	53 (50.5%)	52 (49.5%)		

Abbreviations: ADHD, Attention Deficit Hyperactivity Disorder; ASD, Autism Spectrum Disorder; ASQ, Acorn Satisfaction Questionnaire; CI, confidence interval; CGAS, Children’s Global Assessment Scale; HoNOSCA, Health of the Nation Outcome Scales for Children and Adolescents; *M*, mean; *n*, number; NS, not significant; OR, odds ratio; SD, standard deviation.

Statistical significance: *p < 0.05, **p ≤ 0.01, ***p ≤ 0.001.

**Table 3.** Number of children engaging in different self-harm/suicidal acts during admission (*N* = 69).

Variable	*N*	%
Method of self-harm		
Hitting/punching	50	72.5
Scratching/cutting	48	69.6
Head-banging	45	65.2
Slapping face	29	42
Biting	25	36.2
Self-pinching	17	24.6
Pulling hair	10	14.5
Eating/swallowing objects	10	14.5
Self-strangulation/asphyxiation	9	13
Restricting food	6	8.7
Opening old wounds	5	7.3
Ligatures	4	5.8
Attempted hanging^a^	3	4.4
Other^b^	9	13
Setting		
In front of others	55	79.7
In private	51	73.9
Place		
On the ward	60	87
On leave (home)	46	66.7
Time during admission		
First half	60	87
Second half	37	53.6
Total	69	100

a All incidents occurred at home.

b Refers to deliberate or impulsive acts with an intent to harm oneself, such as running off, walking into traffic, throwing oneself down the stairs/floor, locking oneself in rooms, and climbing/jumping out of windows.

### Variables associated with self-harm and suicidality

Self-harm thoughts were associated with a history of self-harming behavior (*χ* ^2^ = 22.034, OR = 7.86, *p* < 0.001), diagnosis of ADHD (*χ* ^2^ = 4.067, OR = 2.54, *p* = 0.044), longer admissions (*t*(103) = 2.465, OR = 1.01, *p* = 0.015), and at a trend level with history of mental illness in the family (*χ* ^2^ = 3.641, OR = 2.27, *p* = 0.056), and diagnosis of ASD (*χ* ^2^ = 3.607, OR = 2.2, *p* = 0.058). In a separate analysis, engagement in self-harm behavior alone was also significantly associated with previous self-harm (*χ* ^2^ = 21.588, OR = 6.67, *p* < 0.001).

There was a strong association between suicidal ideation and history of self-harm (*χ* ^2^ = 11.042, OR = 4.12, *p* = 0.001), self-harm thoughts (*χ* ^2^ = 38.922, OR = 22.95, *p* < 0.001), and self-harm acts (*χ* ^2^ = 34.568, OR = 16.13, *p* < 0.001) during admission. Diagnosis of ID was negatively associated with suicidal ideation (*χ* ^2^ = 7.309, OR = 0.15, *p* = 0.007). No other factors were significantly associated with self-harm or suicidality (Tables 1 and 2).

### Outcome measures and risk assessments

Statistically significant improvement in outcome measures scores and risk assessment ratings between admission and discharge were identified in our total sample (Table 4). Children in the self-harm group had significantly lower mean CGAS scores on discharge, lower mean CGAS score change, and higher mean HoNOSCA scores on admission and at discharge compared to the non-self-harm group (Table 1). This was not the case for children in the suicidal group vs. the non-suicidal group.

**Table 4.** Outcome measures and risk assessments in the whole sample (*n* = 105).

Variable	Admission *M* (SD)	Discharge *M* (SD)	Change *M* (SD)	SE	95% CI	Significance (two-tailed)
CGAS	29.9 (11.2)	58.3 (13.4)	28.4 (16.8)	.64	[25.1, 31.6]	<0.001
HoNOSCA	21 (6.8)	10.8 (6.2)	−10.2 (7)	.72	[−8.8, −11.6]	<0.001
Self-harm risk^a^	2.28 (1.2)	1.72 (1)	−0.55 (1.2)	.11	[−0.33, −0.78]	<0.001
Suicide risk^a^	1.99 (1.2)	1.42 (0.7)	−0.57 (1)	.10	[−0.38, −0.77]	<0.001

Abbreviations: CGAS, Children’s Global Assessment Scale; CI, confidence interval; HoNOSCA, Health of the Nation Outcome Scales for Children and Adolescents; *M*, mean; SD, standard deviation; SE, standard error

a According to each child’s structured risk assessment.

### Self-harm and suicidality course during admission

**Figure 1.**
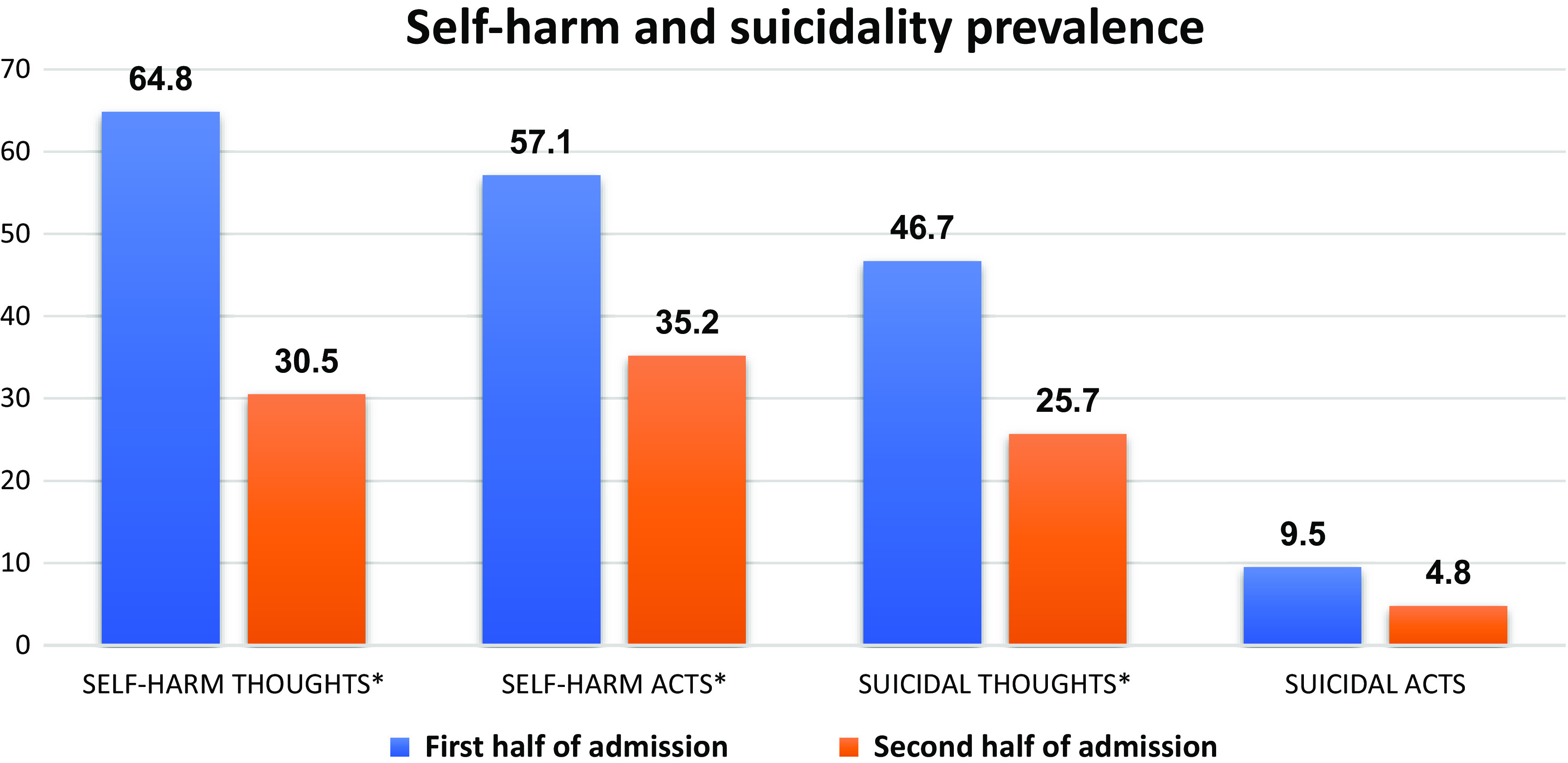
Prevalence (%) of self-harm and suicidality during the first half and the second half of children’s admission in the unit. Statistical significance, *p < 0.001.

Self-harm thoughts (*χ* ^2^ = 20.799, *p* < 0.001) and acts (*χ* ^2^ = 23.958, *p* < 0.001) significantly reduced from the first half to the second half of the admission (Figure 1). Only four children who did not self-harm during the first half, harmed themselves during the second half of their admission. Children were also more likely to report suicidal thoughts during their first half of admission (*χ* ^2^ = 21.667, *p* < 0.001).

## Discussion

### Prevalence of self-harm and suicidality

To the best of our knowledge, this is the first study examining separately both self-harm and suicidal thoughts and acts in children up to the age of 12 admitted to a mental health inpatient unit, also providing comparisons with children without these symptoms during their admission. In relation to self-harm, we found that 65.7% of our sample reported self-harm thoughts and 61% harmed themselves during their admission. This rate is significantly higher than some previous studies on hospitalized adolescents; for example, Zhand and colleagues [47] reported a total self-harm prevalence rate of 14%, whilst Phillips and colleagues [61] found that 16.1% of adolescents injured themselves during the first month of their admission. However, there have also been studies reporting much higher rates too, which seem to be similar to ours [16,18,36]. In young children specifically, our finding on self-harm acts also seems to support previous evidence from the study by Palmer and Martin [44] which identified that just over half of all children up to the age of 14 years admitted to hospital engaged in self-harm. Our study adds weight to this finding even in younger ages suggesting that children requiring a mental health inpatient admission commonly present with self-harm, possibly more so compared to some groups of adolescents.

In relation to suicidality, 50.5% of our sample expressed suicidal thoughts and 14.3% engaged in at least one suicidal act during their admission. Our identified high rate of suicidality is consistent with some studies including adolescent samples [16,22,37]. Suicidal thoughts in our sample seem to be more frequent compared to other studies examining this in inpatient children and there seems to be a trend of increasing prevalence over time [37,62–64]. Suicidal acts in our sample are significantly higher compared to the rate of 4.2% reported by Haavisto and colleagues [37] but lower than the rate of 24% reported by Asarnow and Carlson [51] in a small study including 25 children. This may be related to differences in clinical characteristics between studies. Indeed, the former study included higher functioning children and young people, as evidenced by their CGAS scores, many of whom also had conduct disorders, and the latter included larger numbers of children with major depression (44%) and conduct disorder (44%). It may be less reliable to directly compare the prevalence of suicidal acts to other older studies as the reported rates of “mild suicidal attempts” are very high and in some studies higher than those of “suicidal ideation” which seems to indicate that some instances of non-suicidal self-injury may have been included under this category.

### Risk factors associated with self-harm and suicidality

It is notable that there were no significant correlations between gender and self-harm or suicidality in our sample. Previous studies reported a female predominance among inpatient adolescents who engaged in self-harm or suicidal acts [37,47,61,65]. However, in children these differences are less clear. Palmer and Martin [44] reported a similar pattern in children up to the age of 14, that is a slightly older age group than ours, with females self-harming more commonly than males. However, Haavisto and colleagues [37] identified similarly high prevalence of suicidal ideation in boys and girls up to the age of 12 (42.9 vs. 43.5%) and apparently higher prevalence of suicidal attempts in boys compared to girls (4.9 vs. 2.2%) in this age group. In that study, suicidal attempts in adolescent girls aged 13–18 were strikingly more common than in boys (25 vs. 6.5%) which seems to be driving the statistically significant higher rates of suicidal attempts in girls in the whole group. Children’s gender did not seem to be associated with reported suicidal ideation, apparently including self-harm thoughts, in the study by Fite and Colleagues [50], with suicide risk in the study by Becker and Colleagues [48], or with suicide attempts in the study by Bodzy and Colleagues [49]. Taken together, these findings indicate that self-harm and suicidality in children up to the age of 12 requiring inpatient admission in recent years is not associated with gender which is in line with earlier studies [62,66].

In our sample, age on admission was also not identified as a significant factor associated with self-harm and suicidality. De Kloet and Colleagues [18] suggested that inpatients over 11 years were 10 times more likely to harm themselves than younger children. Within the younger age group of hospitalized children aged 5–13, Myers and Colleagues [63] reported that suicidality increased with age. This seems to be driven mostly by children aged 5–7 years being under-represented in the group of all suicidal children (11.4%), compared to 8–10-year-olds (41%) and 11–13-year-olds (47.4%). As the number of all admitted children in each age bracket is not available, these results are not directly comparable with ours, and may misrepresent actual prevalence rates of suicidal acts in admitted children within their respective age groups. Age was not associated with increased or more severe suicidality in a number of other studies [48,49,51].

In relation to diagnosis, ADHD and ASD were prominent among self-harming children in our sample. Highly distressed children with these conditions may have reduced insight into their emotions and understanding of their frustration which may result in internalizing their difficulties and experiencing self-harm thoughts. Our findings did not support an association between anxiety disorders or depression and self-harm or suicidality. No study so far has identified a link between anxiety disorders and self-harm in children admitted to a mental health unit. In relation to depression, previous studies on hospitalized children directly examining this found association with suicidality [63,64,67]. The difference in our findings may be affected by sample characteristics; our sample included a large proportion of children with neurodevelopmental disorders and no children with conduct disorders. Notably, we found that children with ID were less likely to report suicidal thoughts. This may be related to their difficulty in recognizing or communicating their distressing thoughts, and staff members possibly struggling to assess the nature and intent of their behavior.

Self-harming children stayed in the hospital, on average, for 59 days more than the non-self-harming children. Previous evidence indicated that short admissions can be as effective as longer ones, or even more beneficial [68,69]. Haavisto and Colleagues [37] observed that inpatient treatment of children and adolescents over 90 days was associated with suicidal ideation (OR 1.6) and suicidal attempts (OR 2.0), the latter also being most notable in girls (OR 3.9). By contrast, a study from Australia found that self-harming adolescents who stayed longer in a residential program compared to non-self-harming adolescents (11.3 vs. 8.9 weeks), reported significantly better clinical outcomes [65]. The design of our investigation did not allow conclusive exploration as to the direction of this association in children. That is, whether children who present with self-harm thoughts and behaviors remain as inpatients for longer until this risk is mitigated, or longer admissions may have a negative effect on self-harm. The fact that self-harm overall decreased during admission is probably indicative of the former.

Children with history of self-harm behavior were almost eight times more likely to communicate self-harm thoughts, over six times more likely to injure themselves, and over four times more likely to express suicidal thoughts during their admission. Moreover, children in the suicidal group were almost 29 times more likely to express self-harm thoughts and 16 times more likely to engage in self-harm during admission compared to the non-suicidal group. This is consistent with previous findings indicating a strong association between history of self-harm and recent self-injurious or suicidal behavior in hospitalized children and young people [47,66].

Finally, an interesting finding was that over half of the self-harming children engaged in self-injurious acts while on leave from the inpatient unit. This underscores the importance of carers’ involvement in risk recognition and safety planning. Co-producing crisis and safety plans with patients and carers, before the child leaves the unit, and providing regular support or contact from ward staff while the child stays at home, could support smooth transitions, wider systemic and holistic approaches, and promote self-harm and suicide prevention. In this context, caregivers can improve safety strategies by participating actively in parental training programmes, joint risk assessments, and family therapy groups.

### Risk assessment ratings and outcome measures

Both self-harm and suicide risk assessment ratings were significantly decreased during admission for all children, indicating that the inpatient treatment was beneficial for self-harm and suicidality in this age group. Importantly, there did not seem to be an escalation of self-harm on average due to copying behaviors from admission to discharge in this age group. Additionally, there were no significant differences in service user and parental satisfaction between self-harming/suicidal and non-self-harming/non-suicidal children. This indicates that the needs of all patients were equally met, regardless self-harm or suicidality status.

### Limitations

The main limitations of our study are related to its retrospective design. Its nature did not allow for conclusions on individual self-harm or suicidal incidents and their triggers, nor on the potential contribution of adverse childhood experiences or specific family history of such incidents on childhood self-harm/suicidality. It was not possible to examine the actual number or intensity of all self-harm and suicidal acts during admission or to consider other situational factors, such as medication, staff approaches, or therapeutic interventions. In addition, clinical risk assessments have an element of subjectivity and the administration of validated self-harm/suicidality scales was not possible. Finally, some data were missing.

### Conclusion

Self-harm and suicidality were very common among children admitted into a mental health inpatient unit. A history of self-harm predicted self-harm and suicidality, whilst suicidality appreciably overlapped with and predicted self-harm in this population. The prevalence of self-harm and suicidal thoughts and acts was significantly reduced during admission. Non-self-harming children seem to have marginally better functional outcomes upon discharge compared to self-harming children. Both children and parents/caregivers were found to be similarly satisfied from the inpatient treatment, irrespective of child’s self-harm or suicidality status. Better understanding of the mechanisms and factors related to self-harm and suicidality in this age group, and risk-focused training for healthcare professionals involved in the care of children are paramount in minimizing risk and facilitating positive changes.

## Data Availability

The data that support the findings of this study are available from the authors. Restrictions in relation to potentially person identifiable information apply.
